# The Influence of Ageing and Hydrothermal Fatigue (Thermocycling) on Degradation and Fracture Toughness of Light-Cured and Hybrid Resin-Based Nanocomposites (RBCs)

**DOI:** 10.3390/jfb17060276

**Published:** 2026-06-02

**Authors:** Daniel Pieniak, Agata Maria Niewczas, Agata Walczak, Jarosław Selech, Dorota Czarnecka-Komorowska, Jonas Matijošius

**Affiliations:** 1 Faculty of Safety Engineering and Civil Protection, Fire University, 52/54 Słowackiego Street, 01-629 Warsaw, Poland; dpieniak@apoz.edu.pl (D.P.); awalczak@apoz.edu.pl (A.W.); 2 Faculty of Medical Dentistry, Medical University of Lublin, 19 Chodźki St., 20-093 Lublin, Poland; agata.niewczas@umlub.edu.pl; 3 Faculty of Civil and Transport Engineering, Institute of Machines and Motor Vehicles, Poznan University of Technology, 3 Piotrowo St., 60-965 Poznan, Poland; jaroslaw.selech@put.poznan.pl; 4 Faculty of Mechanical Engineering, Poznan University of Technology, 3 Piotrowo St., 60-965 Poznan, Poland; dorota.czarnecka-komorowska@put.poznan.pl; 5 Mechanical Science Institute, Vilnius Gediminas Technical University, Plytinės Str. 25, 10105 Vilnius, Lithuania

**Keywords:** resin-based composites (RBCs), thermocycling, hydrothermal ageing, fracture toughness (K_IC_), work of fracture (WOF)

## Abstract

The aim of this study was to evaluate the influence of artificial saliva ageing and cyclic hydrothermal loading on the mechanical properties of dental composite materials. Two commercial composites (Filtek Z550 and Filtek Ultimate Flow) and two experimental materials representing flow-type and hybrid composites were investigated. SENB specimens were prepared in accordance with ASTM E399, together with flat specimens intended for impact strength testing using the Dynstat method. All samples were aged in artificial saliva for approximately one month at 37 ± 1 °C, and subsequently, half of the specimens were subjected to thermocycling in the temperature range of 10–65 °C for 10,000 cycles. Static mechanical tests, including three-point bending (TFS), biaxial flexural strength (BFS), and compression strength (CS), were performed before and after thermocycling. In addition, impact strength and fracture toughness expressed by the stress intensity factor K_IC_ were determined. The results were analyzed in terms of the residual work of fracture (WOF), while the durability of the materials was evaluated using Weibull distribution parameters. The experimental analysis was complemented by SEM observations of the microstructure. The obtained results demonstrated a pronounced deterioration of mechanical properties after hydrothermal loading. The average impact strength after artificial saliva ageing reached 11.69 J/mm^2^ for Filtek Z550, 11.57 J/mm^2^ for Ex-hyb(P), 16.39 J/mm^2^ for Filtek Ultimate Flow, and 10.27 J/mm^2^ for Ex-flow(P), whereas after thermocycling, these values decreased to 5.38 J/mm^2^, 8.86 J/mm^2^, 4.55 J/mm^2^, and 4.39 J/mm^2^, respectively. A similar trend was observed for the fracture toughness parameter KIC, which decreased considerably after thermocycling for all investigated materials. The analysis of the residual work of fracture revealed the influence of thermocycling on the energy-related parameters of the composites. In the case of TFS, the average WOF decreased, among others, from 13.65·10^−3^ J to 1.90·10^−3^ J for Filtek Ultimate Flow and from 4.76·10^−3^ J to 2.37·10^−3^ J for Filtek Z550. For BFS, a noticeable decrease in WOF was also observed, particularly for Ex-flow(P) and Filtek Ultimate Flow. In the compression tests (CS), the changes were less unambiguous, and some materials exhibited an increase in WOF after thermocycling. Furthermore, changes in the scale and shape parameters of the Weibull distribution were identified, indicating degradation of composite durability under hydrothermal loading. The results confirmed that cyclic hydrothermal loading exerts a greater influence on impact strength and fracture toughness than on static flexural strength. While all investigated materials exhibited degradation, the extent of changes was material-dependent, and compression behaviour showed non-uniform responses. Weibull analysis confirmed reduced reliability and increased heterogeneity of the composites after ageing, indicating that hydrothermal fatigue is a dominant factor governing long-term mechanical deterioration of dental resin-based composites.

## 1. Introduction

Resin-based composites (RBCs) are widely used in restorative dentistry as filling materials. In everyday dental practice, they are very often the material of first choice for treating dental caries. The oral environment is complex and variable due to the presence of saliva, changing pH levels, and biomechanical and thermal stresses. RBC fillings must be resistant to the changing conditions and factors of the oral environment [1,2]. During the chewing act (cycle), the dynamics of mechanical loads are variable, and the forces acting on a tooth with a filling range from 110 to 250 N and 390–900 N. Initial forces range from 10 to 20 N during normal chewing and from 50 to 150 N in the final phase of the cycle [3,4,5]. Maximum vertical bite forces can reach up to 1000 N [6].

The second type of stress is thermal stress. Consuming cold or hot liquids and foods causes temperature changes in the area surrounding the dental filling [7,8]. The typical temperature range for tooth surfaces is 1–50 °C [9,10,11]. Heating of the filling material (composite) occurs mainly in contact with liquids, and thermal gradients cause stresses to form in the filling material, the distribution of which depends, among other things, on the phase structure of the composite and the filler nanoparticles [12]. Thermal loads can be slow, isothermal, with temperature stabilization within the composite volume over time, or sudden, in the form of thermal shock [13]. The cumulative effects of the oral environment significantly reduce the material’s reliability [14]. Degradation mechanisms have already been described in polymer composites exposed to UV-A radiation and thermal shocks, where cumulative environmental effects significantly reduce material reliability [15]. Thermal loads may be either isothermal or abrupt (thermal shock) [16], and their effects are simulated under laboratory conditions through thermocycling, which—despite simplifying real-world conditions—provides valuable preclinical data [17,18,19,20,21].

The durability of dental filling materials is a key criterion for their clinical success [22]. The literature emphasizes that fillings can function for an average of 4 to 8 years [23], which corresponds to approximately 10^5^–6·10^5^ thermal cycles during that period [24,25].

From a clinical point of view, the durability of direct composite restorations, in both anterior and posterior teeth, depends on many factors. The World Dental Federation has approved new clinical criteria. It has divided them into three groups: esthetic, functional, and biological criteria [15]. In the study, the authors presented research findings related to the second group of criteria, which describes

(a) the presence of cracks in the filling material,

(b) filling fractures,

(c) surface quality.

Many researchers attribute the fracture of a filling part as the main cause of reduced longevity. Other studies point to cracks within the filling and polymerization-induced deformation of the filling mass as the main causes leading to marginal leakage [15,16,17,18]. In clinical assessment, a rough, cracked surface is unacceptable for both functional and esthetic reasons. Such a surface promotes the formation and retention of dental plaque. The presence of dental plaque and microbial biofilm causes demineralization of hard dental tissues, dental caries, pulp diseases, and periodontal diseases [19,20,21]. Clinical studies provide a reliable assessment of composite dental fillings. However, these studies are long-term, and the dentist evaluating the filling is unable to unequivocally identify which oral environmental factor had a decisive influence on the degradation of the filling material. Hence, there is a need for in vitro studies. In vitro, laboratory studies provide only an approximation of real conditions. However, they provide valuable preclinical data. They also allow for the assessment of the impact of individual factors in the oral environment on the filling material [26,27,28].

RBCs undergo degradation due to material fatigue caused by cyclic mechanical and thermal loads [29]. This process leads to the formation of microcracks under subcritical conditions, which is a common cause of premature clinical failure [30]. Degradation of the RBC structure occurs mainly as a result of two mechanisms: hydrolytic ageing and thermal fatigue [31,32]. Hydrolytic ageing is more pronounced in the presence of cyclic thermal and mechanical loads, as tensile stresses promote the formation of microdamage and facilitate water penetration [33,34]. In the case of multiphase composites, including those containing powder fillers, differences in the thermal expansion coefficients of the matrix and the filler particles cause thermal stresses [35]. These differences lead to the formation of thermal gradients and stresses in the material, depending on the geometry of the filling [36].

An important degradation factor is the difference in the coefficients of thermal expansion between the resin matrix and the filler particles. For RBC resins, the coefficient α ranges from 110.1 to 173.8·10^−6^/°C, whereas for selected microhybrid composites, it ranges from 23.2 to 33.0·10^−6^/°C, and for the nanohybrid composite Filtek Supreme, it equals 50.8·10^−6^/°C [12,37]. These differences lead to the formation of first- and second-order thermal stresses as well as temperature gradients within the material [37]. These phenomena also depend on the geometry and class of the dental restoration [38].

The durability of composite materials is considered one of the fundamental criteria for evaluating their clinical applicability [39,40]. Studies indicate that composite dental restorations remain functional for an average period of 4–8 years [41]. During this time, the material is exposed to intensive hydrothermal loading; according to various authors, between 10^5^ and 6·10^5^ thermal cycles occur during 5 years of clinical service [42,43,44].

The energy-related properties, such as impact strength, crack initiation resistance under plane strain (KIC), and work of fracture (WOF), are crucial for assessing the mechanical strength of RBCs. Impact strength reflects a material’s ability to absorb energy under sudden loading, which is important in the context of accidental mechanical trauma in the oral cavity [35,45]. KIC allows for the assessment of a material’s tendency to propagate microcracks, while WOF provides information on the energy required for complete specimen failure, taking into account both the destructive force and the material’s deformability [46]. The authors of the study assume that these parameters may be particularly useful in assessing the residual durability of dental composites after exposure to degrading factors that simulate normal physiological conditions in the oral cavity.

The parameters WOF (work of fracture), KIC (stress intensity factor at fracture), and impact strength are of key clinical importance because they describe the resistance of resin-based dental composites (RBCs) to crack initiation, propagation, and catastrophic failure under conditions resembling the oral environment. KIC reflects the material’s resistance to the growth of existing microcracks under masticatory loading, which is essential for predicting restoration longevity and preventing sudden brittle fracture [30]. Impact strength is particularly relevant for restored teeth, as most failures of restored teeth occur due to new traumatic events [45,47]. Therefore, higher impact strength may contribute to increased longevity of dental restorations [47]. It is also closely related to filler dispersion within the composite structure. Increased filler loading can improve mechanical properties, while reducing filler size at a constant filler content may enhance flexural strength; however, deformability and impact strength may decrease due to particle agglomeration, reduced homogeneity, and increased rigidity of the material [48].

WOF describes the total energy required to fracture the material and represents its ability to dissipate energy during crack propagation, which is critical for resistance to unstable, catastrophic failure. In clinical conditions, all three parameters are strongly associated with the durability of restorations subjected to cyclic mechanical and hydrothermal loading, which promotes fatigue damage and degradation at the resin matrix–filler interface [12,48]. Consequently, these parameters provide more clinically relevant information on long-term performance than conventional static strength measures alone.

Despite extensive research on hydrothermal degradation of resin-based composites, there is still limited comparative evidence linking fracture-related parameters with reliability analysis across different composite classes under identical ageing conditions. In particular, combined evaluation of impact strength, fracture toughness (K_IC_), and work of fracture (WOF), together with Weibull-based reliability assessment after artificial saliva ageing and thermocycling, remains insufficiently addressed. Moreover, few studies integrate these energy-based fracture metrics with microstructural observations to explain degradation mechanisms across universal, flowable, and experimental composites. This represents a clear gap in understanding how hydrothermal fatigue affects both energy dissipation capacity and failure reliability of modern dental composites under clinically relevant conditions.

The aim of this study was to directly evaluate the effect of cyclic hydrothermal loading (artificial saliva ageing combined with thermocycling) on the mechanical performance and fracture resistance of selected resin-based dental composites, including universal, flowable, and experimental materials. The analysis focused on changes in impact strength, fracture toughness (K_IC_), work of fracture (WOF), and reliability parameters derived from Weibull statistics, in relation to different composite formulations. The null hypothesis (H_0_) assumed that cyclic hydrothermal loading does not significantly affect the mechanical properties, fracture resistance parameters (K_IC_, WOF, impact strength), or reliability of the investigated resin-based composites, regardless of their composition.

## 2. Materials and Methods

### 2.1. Sample Preparation

**Figure 1:**
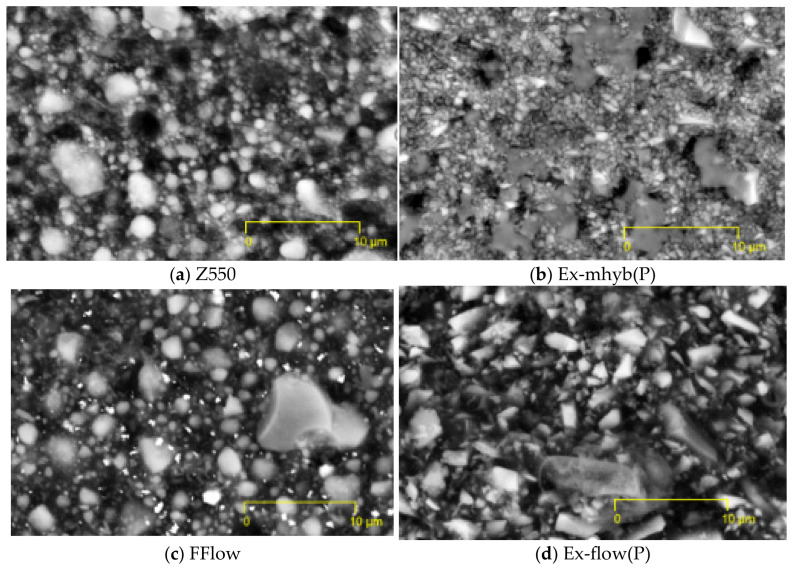
Scanning electron micrographs (5500×) of microstructures for ceramic–polymer dental composites: (**a**) Z550, (**b**) Ex-mhyb(P), (**c**) FFlow, (**d**) Ex-flow(P).

Commercial and experimental polymer and ceramic dental composites (Table 1) were used in the tests. Filtek Z550 (3M ESPE, St. Paul, MN, USA) and Filtek Ultimate Flow (3M ESPE, St. Paul, MN, USA) composites are commercial materials. Filtek Z550 is a nanohybrid composite (Figure 1a) containing clusters of zirconium and silicon nanoparticles and silica nanoparticles. The matrix of Z550 consists of Bis-GMA, UDMA, Bis-EMA, PEGDMA, and TEGDMA resins [49]. According to the manufacturer (3M ESPE), this material has high compression, flexure, and tensile strength, and good abrasion resistance [50]. Filtek Ultimate Flow (FFlow) is a fluid, low-viscosity nanocomposite in which the matrix consists of Bis-EMA, TEGDMA, and Bis-GMA resins [51]. The filler is a combination of 0.1–5 µm particles and silica nanoparticles (20 nm and 75 nm) and zirconium nanoparticle clusters (4–11 nm). The size of the clusters ranges from 0.6 to 10 µm (Figure 1c). Experimental composites were also used in the tests. Ex-mhyb(P) is a microhybrid composite filled with particles of fluorine–barium–aluminum–silicate glass, silicate glass, and titanium oxide (average size 0.90 µm, filler content 79% by weight) (Figure 1b). Ex-flow(P) is an experimental liquid composite with powder filler consisting of fluoride–barium–aluminum–silicate glass, silicate glass, and titanium oxide (average size 0.76 µm, filler content 64% by weight) (Figure 1d). Standardized test specimens were prepared from all materials in accordance with ISO 4049 [52]. The specimens were prepared by a single operator in a split metal mould (+ thin laboratory glass plate) and then light-cured using a Demetron LED curing light (Kerr, Brea, CA, USA). For BFS specimens, the irradiation process was performed using a multipoint approach (ISO 4049 [52]). In the first stage, the central area of the disc-shaped specimen was irradiated, followed by subsequent exposures distributed along the circumference of the specimen. A total of 9 exposure points were applied, with an irradiation time of 40 s per point. All exposures were performed from the top side.

In the case of TFS specimens, irradiation was carried out multipoint-wise along the length of the beam specimen. A total of 5 exposure points were arranged linearly, and each point was irradiated for 40 s. The irradiation direction was from the top side.

Specimens intended for compression testing (CS) were subjected to bilateral irradiation. Two exposure directions were applied, from the upper and lower sides of the specimen. Each side was irradiated for 40 s, ensuring uniform radiation exposure throughout the entire cross-section of the material.

For Dynstat specimens, irradiation was performed multipoint-wise along the longer edge of the specimen. A total of 6 exposure points (2 parallel paths) were applied, with an irradiation time of 40 s per point. The process was carried out from the top side.

In the case of K_IC_ SENB specimens, the irradiation process was also conducted multipoint-wise along the specimen length. A total of 9 exposure points were applied, with an irradiation time of 40 s for each point. All exposures were performed from the top side.

To minimize experimental bias and ensure reproducibility, all specimens were prepared and tested under strictly standardized conditions. Sample preparation was performed by a single operator to eliminate inter-operator variability. All samples were fabricated using the same moulds, curing protocol, and environmental conditions. Mechanical testing procedures were conducted in accordance with relevant international standards (ISO 4049 [52], ISO 6872 [53], ASTM E399 [54], DIN 53435 [55]), ensuring consistency in loading conditions and measurement procedures.

In addition, specimens were randomly assigned to experimental groups (ageing and ageing with thermocycling) to reduce selection bias. All tests were performed using calibrated equipment under controlled laboratory conditions, and identical protocols were applied before and after thermocycling to ensure comparability of results.

### 2.2. Ageing and Thermocycling Procedure

**Figure 2:**
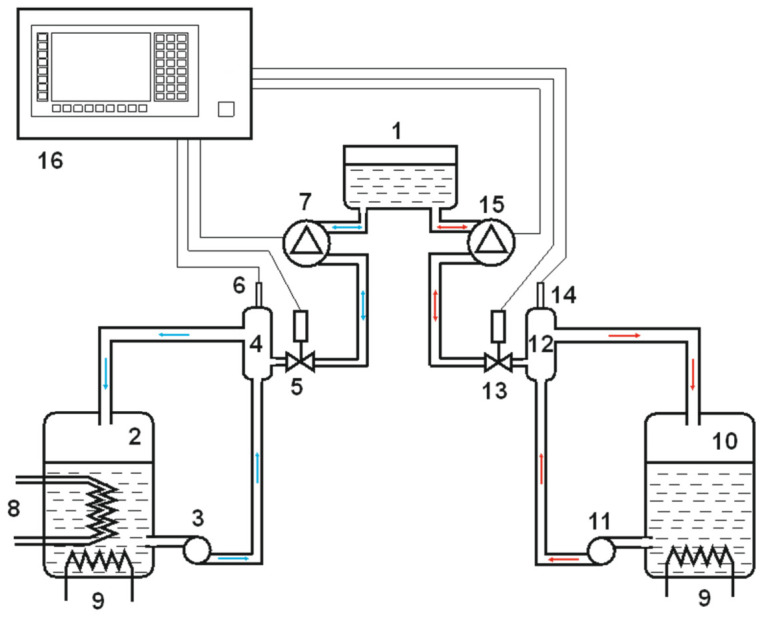
Thermal shock simulator diagram: 1—sample vessel, 2—cooling ultrathermostat, 3—cooling ultrathermostat circulation pump, cooled liquid vessel, 4—cooled liquid vessel, 5—cooled liquid solenoid valve, 6—temperature probe, 7—cooled liquid peristaltic pump, 8—ultrathermostat cooling system, 9—ultrathermostat liquid heaters, 10—heating ultrathermostat, 11—heating ultrathermostat circulation pump, 12—heated liquid vessel, 13—heated liquid solenoid valve, 14—temperature probe, 15—heated liquid peristaltic pump, 16—controller. (The blue arrow indicates the direction of circulation of the cooled fluid. The red arrow indicates the direction of circulation of the heated fluid).

The composite samples were subjected to ageing for two weeks in a Q-Cell 60 laboratory incubator (Pol-Lab, Wilkowice, Poland) in artificial saliva prepared in accordance with ISO 10271 [56], at a temperature of 37 °C ± 0.1 °C. Then, half of the samples were additionally subjected to thermocycling. The choice of an ageing environment in artificial saliva at 37 °C was based on its proven effectiveness in tests simulating conditions in the oral cavity [57]. The thermocycling process consisted of 10^4^ cycles, with a temperature range of 10 °C to 65 °C [58]. The total duration of one cycle was 201 s. The duration of the cycle stages is shown in Table 2. A diagram of the thermal shock device is shown in Figure 2.

### 2.3. Test Methods of Mechanical Properties

**Figure 3:**
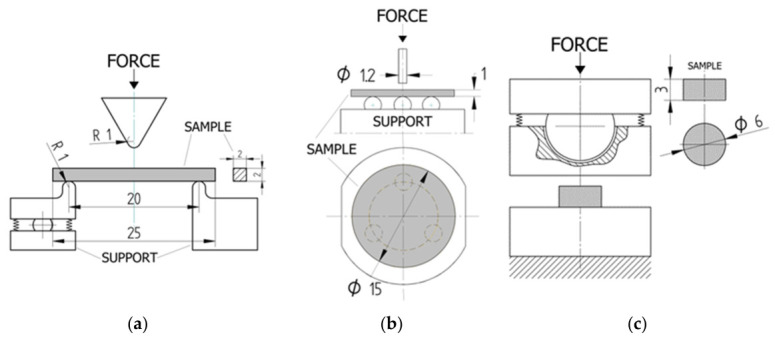
Schematic illustrations of static strength tests: (**a**) three-point flexure strength test, (**b**) biaxial flexure strength test, (**c**) compression test.

The composites were subjected to quasi-static strength tests on aZ100 universal testing machine (Zwick/Roell, Ulm, Germany). A force sensor with a nominal range of 500 N was used in the biaxial flexure tests. The three-point flexure strength (TFS) test was performed in accordance with ISO 4049 [52], according to the diagram shown in Figure 3a. The pressure speed in the three-point flexure test was 0.75 mm/min.

The biaxial flexure strength (BFS) test was performed in accordance with the ISO 6872 [53] technical standard, using the setup shown in Figure 3b. A support consisting of three steel balls (100Cr6) with 120° spacing and a flat loading tip.

The compression strength test was performed using a force sensor with a nominal range of 10 kN, at a pressure speed of 5 mm/min. The measurement system diagram is shown in Figure 3c.

It is known from the literature that in any reconstructive material application involving impact loads, abrasion, or movement of a component, an important characteristic of the material is the amount of energy absorbed to cause damage [59]. In the energy approach to material strength, the work done by external forces causing deformation is equal to the internal energy of the material’s deformation. In testing, this value is determined as the area under the stress–strain curve (σ–ε). The total area of this curve corresponds to the work required to fracture or destroy the material (Work of Fracture, WOF). The WOF value was calculated according to the following equation [60]:

(1) $$WOF=\int_{0}^{y}Pdy\;[Nmm],$$

where *P*—load during test [N], *y*—sample deformation [mm].

The fracture energy is considered one of the most reliable characteristics of a material with a complex internal structure. The work of force during deformation, treated as an energy measure, allows for the assessment of the combined effect of load and deformation on the material. Its threshold value best reflects the degree of damage to the structure of the material.

The critical value of the fracture toughness (*K_IC_*) is an inherent feature of the material and is a measure of its resistance to fracture propagation [61]. The *K_IC_* depends on the external load, fracture depth, and geometric parameters of the sample [62]. Currently, there are many known methods for testing the resistance of dental composites to plane strain fracture [63,64].

**Figure 4:**
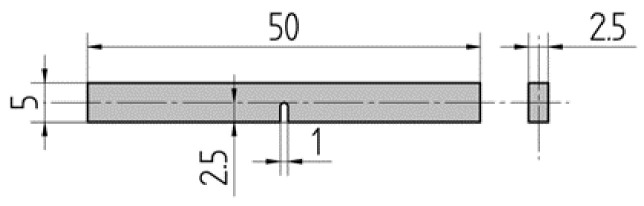
Nominal dimensions of SENB samples used to determine the critical value of fracture toughness *K_IC_*.

In the case of initial cracking according to Griffith’s theory, the K_IC_ value can be determined, among other things, in a three-point flexure test of SENB (Single-Edge Notch Beam) samples [65]. In this study, the critical value of the K_IC_ was determined in accordance with the method contained in ASTM E399 [54]. The tests were performed on cuboid SENB samples with the nominal dimensions shown in Figure 4 (n = 10). The notch was made with an insert placed in the sample-shaping mould. The pressing speed was 0.5 mm/min. The actual dimensions of the notch (a_0_; nominally 2.5 mm) were determined using the optical system of the Vickers Future-Tech FM 700 microhardness tester.

The critical value K_IC_ of stresses for dental composite samples with dimensions and shapes shown in Figure 4 was determined based on the equation:

(2) $$K_{IC}=\frac{PLf_{1}(a)}{bd^{1,5}}\;\left[MPa\cdot m^{1/2}\right],$$

where *P*—load during testing [kN], *L*—support spacing [cm], *b*—sample width [cm], *d*—sample thickness [cm], *a*_0_—notch depth [cm].

The value of *a* parameter was calculated based on the following formula:

(3) $$a=\frac{a_{0}}{d}$$

The *f*_1_ coefficient was calculated based on the equation:

(4) $$f_{1}=\frac{3a^{0,5}\left[1,99-a\left(1-a\right)\left(2,15-3,93a+2,7a^{2}\right)\right]}{2\left(1+2a\right){\left(1-a\right)}^{1,5}}$$

**Figure 5:**
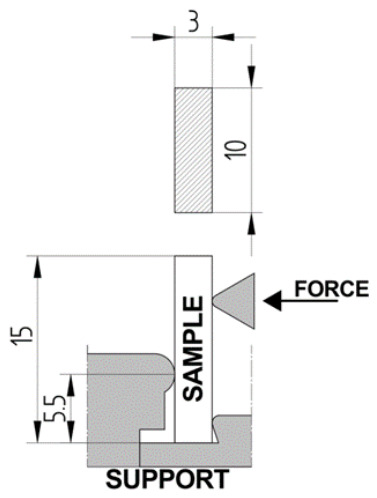
Diagram of Dynstat impact strength test.

The impact strength of dental composites was determined using the Dynstat method in accordance with DIN 53435 [55]. The Dynstat method was used due to the small size of the samples. The tests were performed using a pendulum hammer (Figure 5). Cuboid samples without a notch with dimensions: length *l* = 15 mm, width *b* = 10 mm, and height *h* = 3 mm were used. The impact strength of samples without a notch, *a_n_*, is the work expended to fracture the sample without a notch, relative to the cross-sectional area at the point of fracture:

(5) $$a_{n}=\frac{A_{n}}{b\cdot h}$$

where *A_n_*—work required to fracture the sample [J], *b*—sample width [mm], *h*—sample height at the notch [mm].

The deformability of the sample (Δ*L*) is calculated based on the following formula:

Δ*L* = ((*L*0 − *LF*)/*L*0) × 100% (6)

where *L*0—initial length of the sample before applying load, *LF*—length of the sample after applying load or failure.

The Δ*L* result is expressed as a percentage and determines the relative change in the length of the sample, i.e., the deformability of the material.

### 2.4. Fracture Nature Microscopic Evaluation

In order to assess the nature of the fracture and the mechanism of damage propagation in the tested samples, microscopic analysis was performed using scanning electron microscopy (SEM). Selected samples were thoroughly examined using SEM microscopes FEI Quanta 650 (FEI Company, Hillsboro, OR, USA) and Phenom G2 Pro (Phenom-World B.V., Eindhoven, The Netherlands). SEM analysis allowed for assessment of the morphology of the material structure, the identification of microfractures, and possible structural defects. The resulting images allowed for detailed visualization of hydrothermal damage and the observed defects to be linked to the results of the energy characteristics of the materials.

### 2.5. Statistical Analysis

Statistical analysis was performed using Statistica v. 12.5. Mean values and standard deviations of work of fracture (WOF) were calculated based on the results of strength and impact tests (TFS, BFS, CS) of the analyzed composite samples. The next step of the analysis involved approximating the statistical distribution of the test results using a two-parameter Weibull model in order to estimate the probabilistic prediction of WOF as an energetic measure of composite strength:

(7) $$\lambda \left(W\right)=\frac{m}{W_{0}}\;W^{m-1}$$

where *W*_0_ is a parameter of the Weibull model scale determining the work of fracture of 63.2% of objects in the population; *m* is *a* shape parameter determining the predicted failure intensity curve (and other fatigue strength of the composite).

Depending on the value of parameter m, the intensity of failure (fractures) changes: it increases progressively when m > 2, increases linearly when m = 2, increases degressively when 1 < m < 2, is constant when m = 1, and decreases when 0 < m < 1. The applicability of Weibull reliability estimation has also been demonstrated in systems operating under small-sample constraints, confirming the suitability of this approach for materials where the number of test specimens is inherently limited [66].

**Figure 6:**
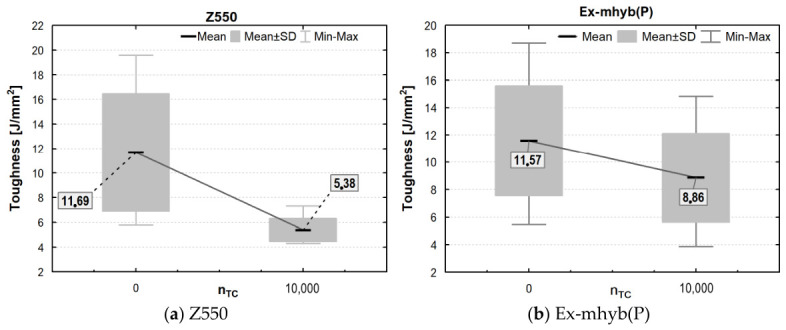

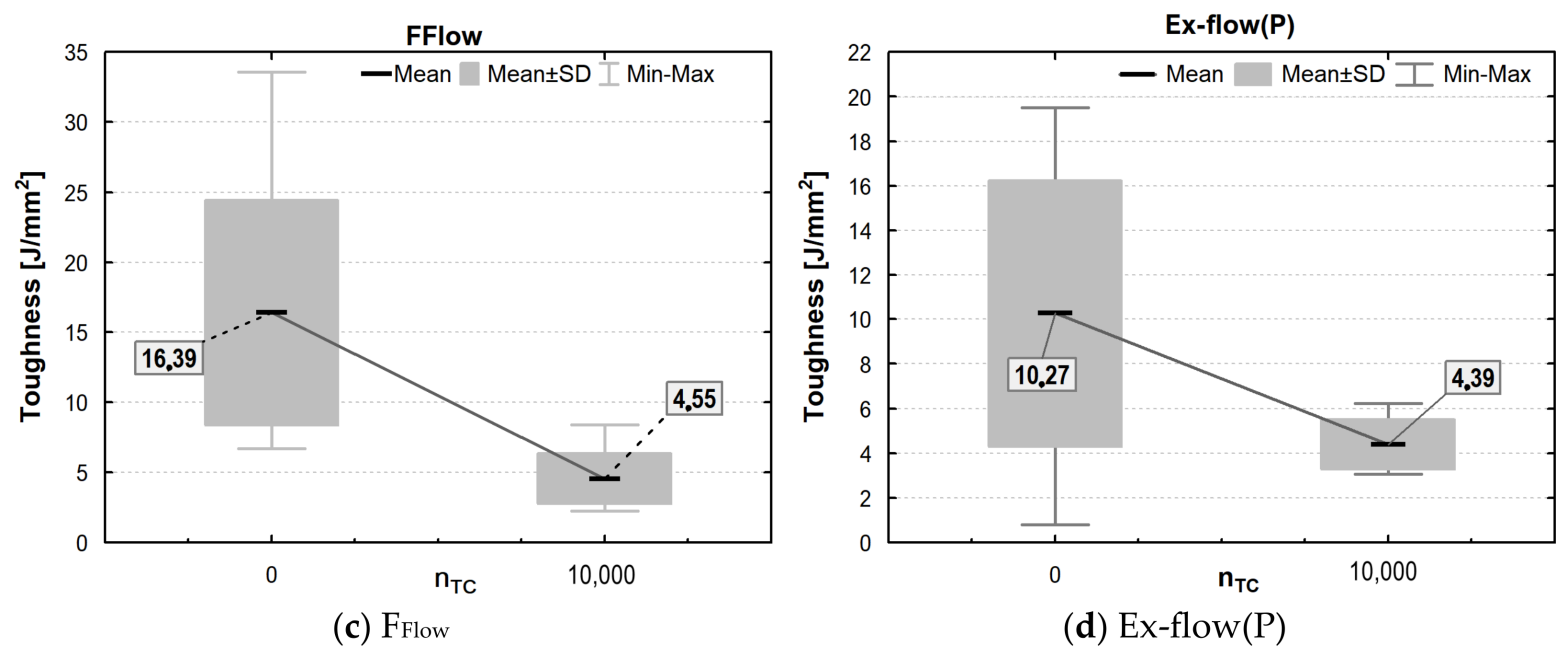
Box-and-whisker plots of impact strength test results: (**a**) Z550, (**b**) Ex-mhyb(P), (**c**) F_Flow_, (**d**) Ex-flow(P).

**Figure 7:**
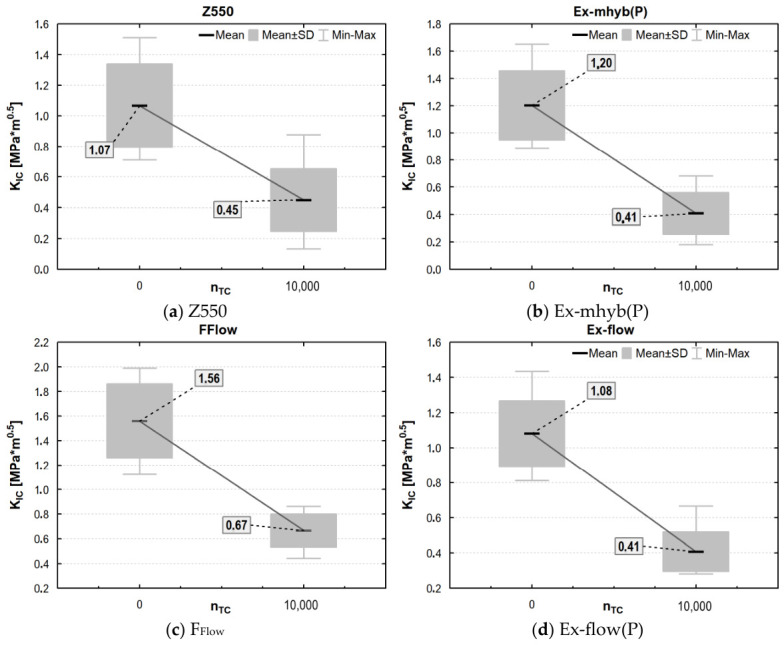
Box-and-whisker plots of critical stress intensity factor *K_IC_* results: (**a**) Z550, (**b**) Ex-mhyb(P), (**c**) F_Flow_, (**d**) Ex-flow(P).

Based on this analysis, the scale parameter W_0_ and the shape parameter m were determined. The methodology was applied according to the approach described in [67]. Results of the impact tests and the critical stress intensity factor (K_IC_) are presented as box-and-whisker plots (Figure 6 and Figure 7, respectively). Mean values, standard deviations, as well as minimum and maximum values, are reported. The effectiveness of Weibull distributions in reliability assessment has also been validated in advanced stochastic degradation models combining Weibull statistics with Wiener processes, demonstrating their broad applicability in evaluating competitive failure mechanisms [68].

The statistical analysis in this study was primarily based on descriptive statistics (mean values and standard deviations) and probabilistic modelling using the Weibull distribution. The Weibull approach was selected due to its suitability for describing the variability and reliability of material strength, particularly in studies involving relatively small sample sizes.

It should be noted that the objective of this study was not to test specific statistical hypotheses but to evaluate trends in material degradation under hydrothermal loading conditions. Therefore, classical inferential statistical tests (e.g., hypothesis testing based on *p*-values) were not the primary focus. Instead, emphasis was placed on the analysis of distribution parameters and comparative trends between material conditions.

The number of specimens used in this study was determined based on commonly accepted practices in mechanical testing of dental composites and relevant international standards. For fracture toughness testing, a sample size of n = 10 is widely considered sufficient to ensure reliable estimation of K_IC_ values under controlled laboratory conditions. For strength and energy-based parameters (WOF, TFS, BFS, CS), larger sample sizes (n = 15–20) were used to improve statistical robustness and allow for reliable estimation of variability using Weibull distribution analysis.

In addition, the application of Weibull statistics, which is specifically designed for the analysis of material strength variability in relatively small datasets, further supports the adequacy of the selected sample sizes. Similar sample sizes have been reported in previous studies on dental resin-based composites, confirming their suitability for comparative mechanical and reliability analyses.

## 3. Results

### 3.1. Mechanical Property Test Results

Mechanical testing revealed substantial differences between compressive and flexural behaviour of the investigated composites, as well as a clear deterioration of mechanical performance after thermocycling. The test results showed that the work of fracture (WOF) in the compression test (CS) was many times higher than in the biaxial (BFS) and three-point flexure (TFS) tests (Table 3). In the TFS and BFS tests, the F_Flow_ material had the highest WOF value after ageing. The lowest initial WOF in the TFS sample was found for the Ex-mhyb(P) material, while the lowest initial WOF in the BFS sample was found for the Z550 material. Materials with a high filler content had the lowest WOF values in both flexure strength tests, suggesting poorer adaptability to tensile stresses.

In flexure strength tests, where destruction was initiated by tensile stresses, a clear decrease in “residual” WOF after thermal cycles was observed for all tested materials. The results indicate that these decreases correlate with reduced deformability of the samples (dL parameter, Table 3). Dental composites exhibit low irreversible deformation and are significantly more brittle than metals [69]. After thermocycling, the deformability of the materials deteriorates further, in some cases by 50% or more. Flow composites exhibited the highest initial deformability in the BFS and TFS tests, while F_Flow_ material exhibited the highest deformability in the CS test. The lowest deformability after thermocycling in the TFS test was recorded for the Ex-mhyb(P) material, and in the BFS test, for the Z550 material, i.e., high-filled composites. In the compression test (CS), the deformability after thermocycling was maintained at a level similar to the initial one, with the greatest change in deformability observed for the F_Flow_ material (Table 3).

The analysis of impact strength results (box-whiskers, Figure 6) confirmed the impact of hydrothermal loads on the impact strength of the tested materials. The remaining impact strength after thermocycling was lower than the initial values and amounted to 76% for Ex-mhyb(P), 46% for Z550, 43% for Ex-flow(P), and 28% for F_Flow_. The lowest impact r strength after thermocycles was observed in flow materials, and the greatest reduction was observed in the commercial F_Flow_ material, which also had the highest initial impact strength. The initial impact strength of high-filled materials (Z550 and Ex-mhyb(P)) was similar.

Similarly to other mechanical parameters, the values of the stress intensity factor K_IC_ had statistical dispersion (Figure 7). The highest initial K_IC_ value was obtained for the FFlow material. For comparison, the manufacturer of reference composites (3M ESPE) provides catalogue values of K_IC_ for Z550 ~2 MPa·m0.5 and for F_Flow_ ~1.6 MPa·m^0.5^ [50,51]. The initial K_IC_ value of the F_Flow_ material obtained in these tests was closest to the catalogue value.

Overall, the results demonstrated that hydrothermal ageing and thermocycling had a pronounced detrimental effect on the mechanical performance of all tested composites, particularly under tensile and flexural loading conditions. The reduction in WOF observed in the TFS and BFS tests was consistently associated with decreased deformability (dL), indicating increased brittleness after thermocycling. Flow-type composites exhibited the highest initial values of WOF, impact strength, and K_IC_, but at the same time showed the greatest susceptibility to hydrothermal degradation, especially the commercial FFlow material. In contrast, highly filled composites demonstrated lower initial deformability and lower WOF values in flexural tests, but their mechanical performance remained relatively more stable after thermocycling. Unlike flexural loading, the compression test results remained comparatively unaffected by hydrothermal ageing, suggesting greater resistance of the composites to compressive stresses than to tensile stresses. These findings indicate that both filler content and composite structure play a key role in determining the resistance of dental composites to hydrothermal degradation.

### 3.2. Results of Microfracture and Other Structural Damage Testing

The microstructural degradation mechanisms of RBCs observed in SEM images are a direct manifestation of fatigue and hydrothermal processes occurring within the material. The key phenomenon is the initiation of microcracks at the resin matrix–filler interface, where water penetration promotes interfacial weakening and stress concentration. Good filler–matrix adhesion may cause crack deflection away from the interface; however, at local surface defects, crack initiation and propagation into the bulk material are clearly observed [29]. In highly filled composites with larger filler particles, the limited deformability of the surrounding matrix promotes stress concentration and the initiation of fatigue microcracks [70].

**Figure 8:**
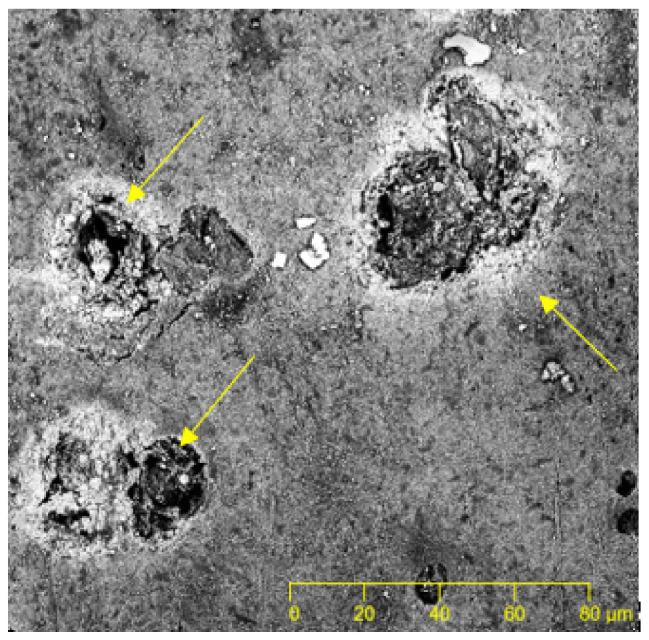
SEM micrograph of damage in the form of surface defects in a Z550 sample material.

**Figure 9:**
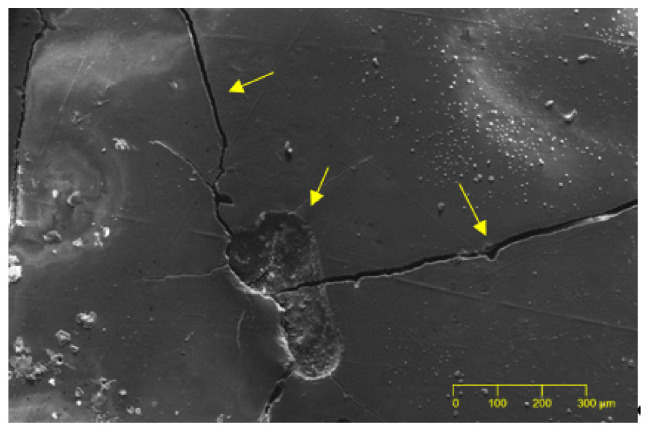
SEM micrograph of surface fracture in Ex-mhyb(P) composite after thermocycling, caused by surface material defect.

**Figure 10:**
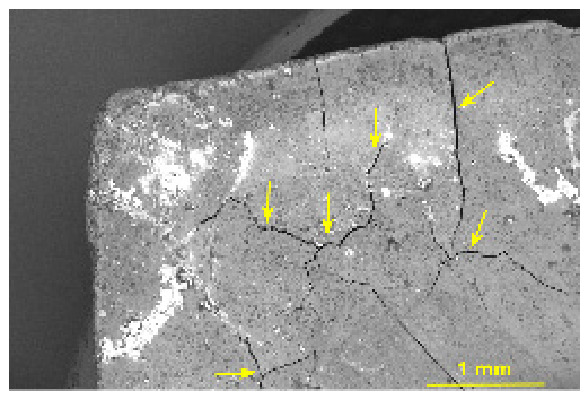
SEM micrograph of the Z550 sample surface after thermocycling, showing the fracture network on the sample surface.

**Figure 11:**
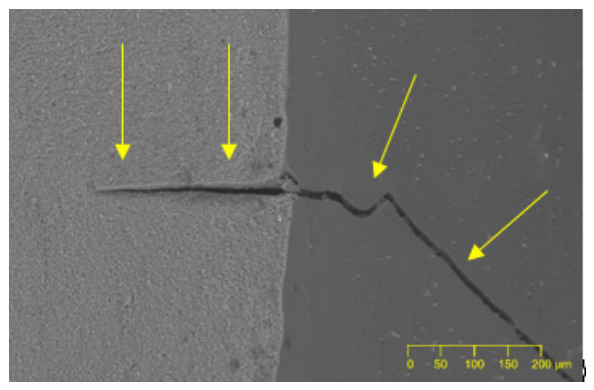
SEM micrograph of Z550 material surface fracture. A fracture extending into the sample volume, visible on the fracture surface of the sample used in impact testing.

SEM analysis revealed surface defects (Figure 8, yellow arrows), which may originate either during material processing or as a result of matrix particle leaching due to fatigue-induced loss of cohesion. After thermocycling, microcracks were observed in Ex-mhyb(P) and Z550 specimens (in Figure 9, Figure 10 and Figure 11 these cracks are marked with yellow arrows), initiating within these surface defects. This indicates that surface imperfections act as local stress concentrators, where the highest hydrothermal stress accumulation occurs, promoting fatigue crack initiation according to the residual stress model $\sigma_{r}=E\cdot ∆\alpha \cdot ∆T$ [44].

**Figure 12:**
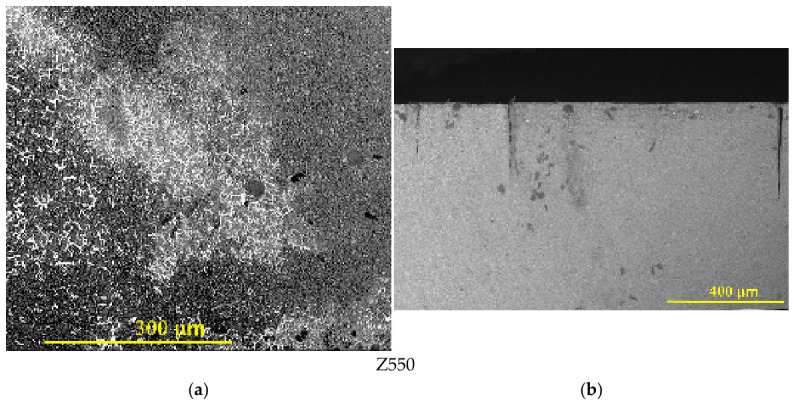

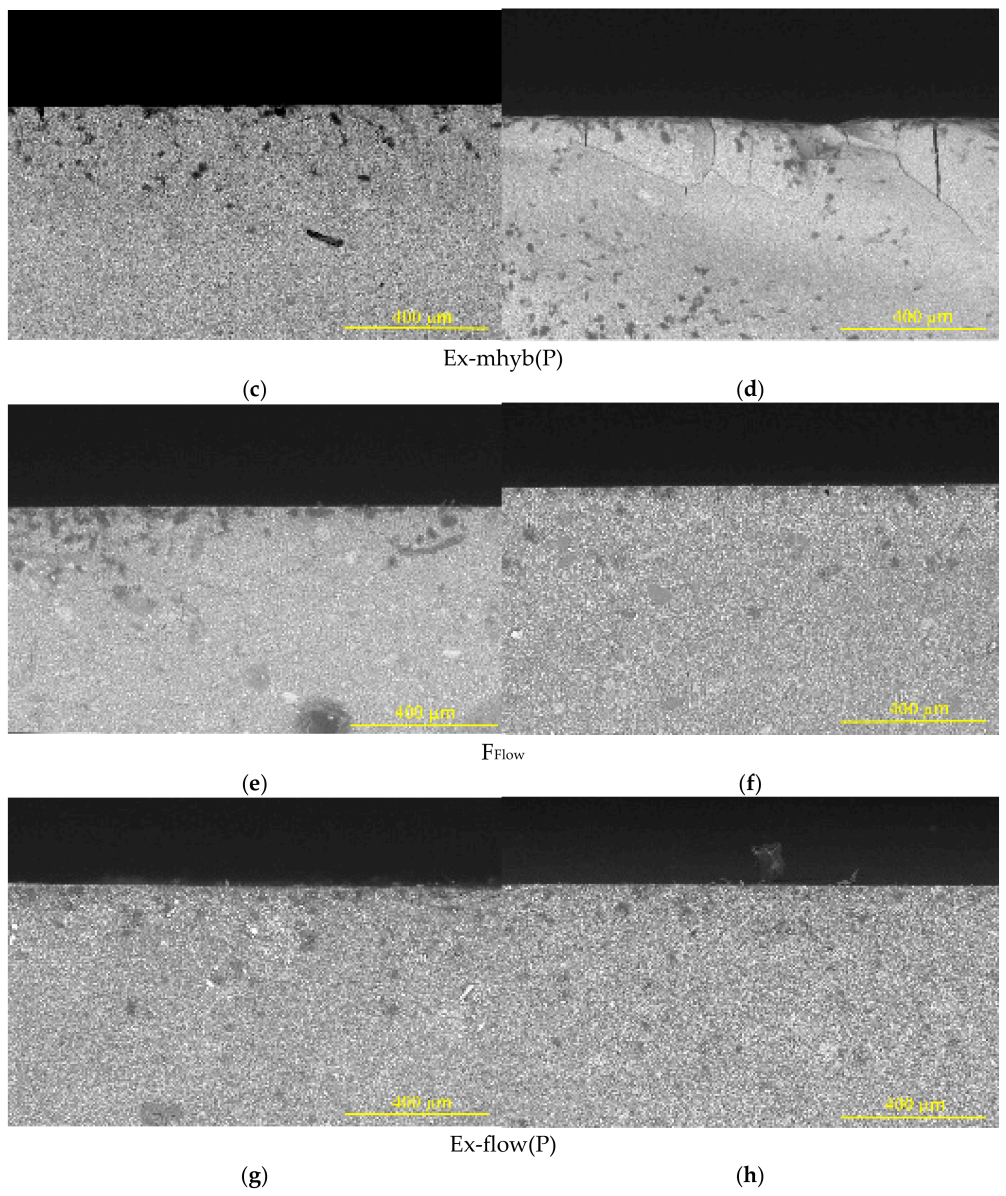
Fracture surface after only ageing and after ageing and 10^4^ thermocycles using the Dynstat test: SEM micrographs, Z550 composite (**a**,**b**); Ex-mhyb(P) composite (**c**,**d**); F_Flow_ composite (**e**,**f**); Ex-flow(P) composite (**g**,**h**); after only ageing (**a**,**c**,**e**,**g**) and after ageing and thermocycles (**b**,**d**,**f**,**h**).

**Figure 13:**
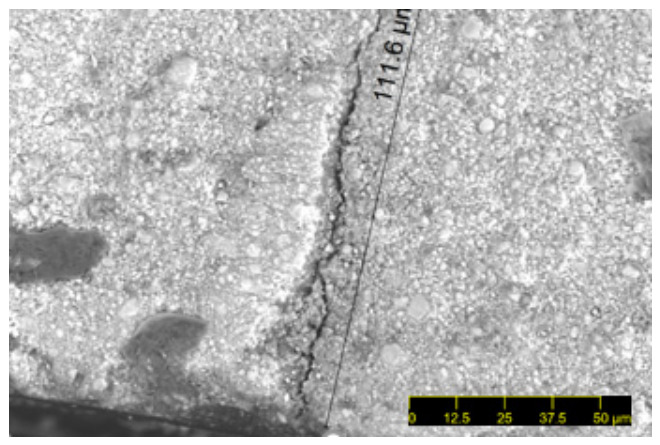
Fracture extending from the surface into the volume of a Dynstat sample after ageing and thermocycling (surface in contact with the conditioned surface at the bottom of the SEM micrograph).

In highly filled composites (Figure 10), a dense network of microcracks (yellow arrows) was observed in the near-surface region, indicating the dominant role of thermal gradients and cyclic liquid exposure in damage development. These cracks propagate from the surface into the material structure (Figure 11, yellow arrows). In flow-type composites, only isolated or no such defects were observed (Figure 12), suggesting a more homogeneous structure and lower susceptibility to crack initiation. In highly filled composites cracks propagated into the material to depths ranging from several tens to over 100 µm (Figure 13), consistent with surface stress intensification described in the literature [45]. 

The SEM observations confirm that the mechanical degradation of RBCs results from the synergistic interaction of three mechanisms: weakening of the interfacial phase, stress concentration at surface defects, and the development of fatigue microcracks induced by cyclic hydrothermal loading [48].

The results of residual mechanical property tests, both static and dynamic, indicate the role of microfractures extending from the surface of the material. However, the observed decreases in mechanical parameters in cases where tensile stresses were predominant may suggest the presence of additional microfractures in the volume of the samples. However, defects of this type are difficult to confirm unequivocally.

**Figure 14:**
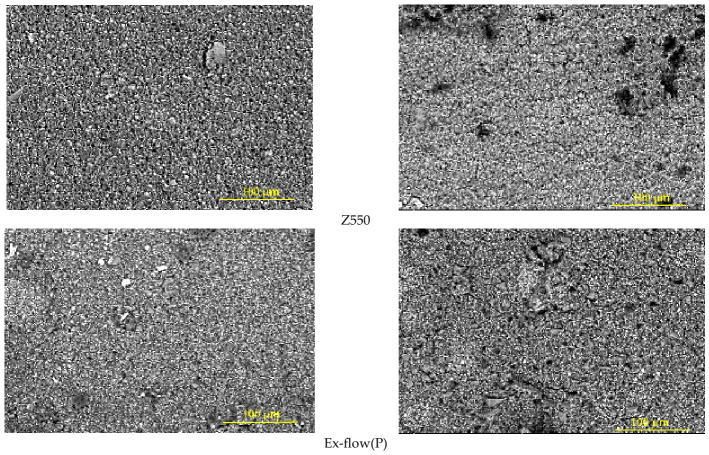
Volumetric damage is visible in cross-sections of tested material samples.

Figure 14 shows SEM micrographs of samples subjected only to ageing (left) and samples after thermocycling (right). The micrographs were made on cross-sections located at approximately ¼ of the sample thickness. The SEM micrographs show microdefects, with their higher density in the samples after thermocycling. These results indicate the presence of fatigue decohesion in the volume of the material, which suggests that the degradation of mechanical properties results from both surface and volume damage.

## 4. Discussion

### 4.1. Main Findings

Hydrothermal degradation of dental composites in the oral environment is a complex process involving cyclic thermal stresses, water sorption, and hydrolytic ageing [70,71,72,73,74,75]. Previous studies have demonstrated that exposure to moisture and thermal cycling reduces the mechanical performance of resin composites, particularly under flexural loading conditions [76,77,78,79,80,81,82,83]. The present results confirm these observations, showing a substantial reduction in work of fracture (WOF), impact strength, and fracture toughness after thermocycling [84,85,86].

The decrease in WOF observed in the TFS and BFS tests indicates that hydrothermal fatigue significantly reduces the energy absorption capacity of composites under tensile stresses. Earlier studies have shown that energy-based parameters such as WOF are more sensitive indicators of degradation processes in composites than conventional mechanical parameters alone [74,75,76]. The degradation observed in the present study was particularly pronounced in flow-type composites, especially FFlow, despite their high initial mechanical performance. These findings suggest that materials with higher initial deformability may also be more susceptible to hydrothermal ageing. In contrast, highly filled composites exhibited lower initial deformability and lower WOF values, but their properties remained relatively more stable after thermocycling.

However, the magnitude of degradation observed in the present study was greater than that reported in some previous investigations [87,88,89,90]. This discrepancy may be related to differences in thermocycling protocols, exposure duration, specimen geometry, and loading configuration. In particular, energy-based parameters such as WOF appear to be more sensitive to microstructural damage accumulation than conventional strength measurements [91,92,93].

Weibull analysis demonstrated that both the shape parameter (m) and scale parameter were affected by thermocycling, indicating changes in structural reliability and fracture behaviour. The highest m values were observed for composites containing smaller filler particles and higher filler homogeneity. This suggests that filler homogeneity and particle distribution may contribute not only to higher initial reliability but also to greater resistance against statistically dispersed crack initiation after hydrothermal ageing [94,95,96]. The observed reduction in characteristic WOF values after thermal cycling confirms that hydrothermal ageing influences not only average mechanical performance but also the statistical distribution of fracture resistance.

Impact strength and fracture toughness (K_IC_) also decreased after thermocycling, confirming the increased brittleness of the composites following hydrothermal degradation. The strongest deterioration was again observed for flow composites. Although FFlow initially exhibited the highest impact strength and K_IC_, its residual properties after thermocycling were among the lowest. These observations are consistent with previous reports indicating that hydrothermal fatigue weakens the resistance of resin composites to crack initiation and propagation [97,98].

It should also be noted that direct comparison of K_IC_ values reported in the literature is difficult because fracture toughness measurements are highly sensitive to specimen geometry, notch preparation, and loading methodology. Nevertheless, the consistent reduction in K_IC_ after thermocycling observed across different studies supports the conclusion that hydrothermal ageing weakens crack propagation resistance in resin composites.

The degradation mechanisms identified in this study are consistent with those described in the literature [99,100,101,102] and include

- hydrolytic weakening of the filler–matrix interface,
- residual thermal stresses caused by differences in thermal expansion between composite components,
- hydrolysis of the polymer matrix and interfacial bonds.

These mechanisms likely act simultaneously, promoting microcrack initiation and progressive structural degradation, particularly in the surface layer directly exposed to thermal and moisture fluctuation.

From a clinical perspective, the observed reduction in WOF, impact strength, and fracture toughness after thermocycling may contribute to increased susceptibility of restorations to crack initiation, marginal deterioration, and bulk fracture during long-term intraoral service. The greater degradation observed in flowable composites suggests that these materials may be more sensitive to hydrothermal ageing when used in stress-bearing posterior restorations. In contrast, the relatively higher stability of highly filled composites after thermocycling may be advantageous in clinical situations requiring long-term resistance to cyclic occlusal loading.

### 4.2. Limitations and Future Research Directions

Despite the comprehensive experimental programme, several limitations of the present study should be acknowledged. First, the number of thermocycles (10^4^) represents a simplified approximation of clinical conditions and does not fully capture long-term intraoral exposure, where thermal and mechanical loads occur simultaneously and interactively. Second, the ageing protocol was limited to artificial saliva under controlled laboratory conditions, which does not reflect the full complexity of the oral environment, including enzymatic activity, pH fluctuations, and biofilm interactions. Third, the study focused on selected commercial and experimental resin-based composites, and therefore, the results cannot be directly generalized to all classes of dental restorative materials.

Another limitation is related to the sample geometry and loading conditions. Standardized specimens were used to ensure repeatability and comparability of results; however, they do not fully represent the complex geometry and stress distribution in clinical restorations. In addition, microscopic observations were primarily qualitative, which limits the possibility of establishing direct quantitative correlations between microstructural damage and mechanical degradation.

Future research should focus on extending the scope of hydrothermal fatigue studies by incorporating combined thermo-mechanical loading, higher numbers of cycles corresponding to long-term clinical service, and more advanced ageing environments that include chemical and biological factors. The application of in situ monitoring techniques, such as acoustic emission or digital image correlation, could provide deeper insight into damage initiation and propagation mechanisms. Furthermore, the integration of experimental results with numerical modelling and probabilistic approaches (e.g., coupled fatigue–reliability models) may improve the prediction of long-term performance of dental composites.

The findings of this study may also be relevant beyond dental materials science. The demonstrated sensitivity of energy-based parameters, such as work of fracture (WOF) and fracture toughness (K_IC_), to hydrothermal degradation can be applied in the broader context of polymer-based composites used in biomedical engineering, micro-mechanical systems, and structural components exposed to cyclic environmental loading. In particular, the approach combining energy-based analysis with Weibull statistics may be beneficial for reliability assessment in engineering applications where material degradation is governed by stochastic processes.

## 5. Conclusions

The aim of this study was to evaluate the effect of cyclic hydrothermal loading under controlled laboratory conditions on selected energy-related mechanical parameters (impact strength, K_IC,_ and WOF) of commercial and experimental dental nanocomposites:

1. Thermocycling in a humid environment reduced the residual mechanical performance of all tested nanocomposites, particularly the work of fracture (WOF) and impact strength. The most pronounced changes were observed under flexural loading conditions (BFS and TFS). Hydrothermal cycles influenced the scale and shape parameters of the Weibull distribution, indicating changes in fracture behaviour and structural reliability that depended on composite composition and filler characteristics.
2. The surface defects and volume damage were observed. Surface defects act as local notches, initiating microfractures, and in high-filled composites, fractures can extend to a depth of >100 µm. The obtained results suggest that both surface defects and volumetric degradation mechanisms contribute to crack initiation and propagation after thermocycling. The test results may have practical relevance; however, their direct clinical applicability is limited by the experimental conditions of the study. The findings provide a basis for further investigations and may support the selection of materials in combination with additional clinical and long-term studies.

## Figures and Tables

**Table 1:** Properties of tested materials.

Property	Filtek Z550	Filtek Ultimate Flow	Ex-mhyb(P)	Ex-flow(P)
Material type	Nanohybrid	Liquid nanocomposite	Microhybrid	Liquid composite
Matrix	Bis-GMA, UDMA, Bis-EMA, PEGDMA, TEGDMA	Bis-GMA, UDMA, Bis-EMA, PEGDMA, TEGDMA	Bis-GMA, UDMA, Bis-EMA, PEGDMA, TEGDMA	Bis-GMA, UDMA, Bis-EMA, PEGDMA, TEGDMA
Filler	Zirconium, silica	Zirconium, silica	Fluoride–barium–aluminum–silicate glass, titanium	Fluoride–barium–aluminum–silicate glass, titanium
Average particle size	0.6–10 µm (clusters)	0.6–10 µm (clusters)	0.90 µm	0.76 µm
Filler content	~78.5% by weight (~63.3% by vol.)	~78.5% by weight (~63.3% by vol.)	79% by weight	64% by weight
Compression strength	~385 MPa	~385 MPa	No data available	No data available
Flexure strength	~150 MPa	~150 MPa	No data available	No data available
Tensile strength	~85 MPa (Brazilian)	~85 MPa (Brazilian)	No data available	No data available
Abrasion resistance	Linear wear ≈ 5 µm/2·10^5^ cycles	Linear wear ≈ 5 µm/2·10^5^ cycles	No data available	No data available

**Table 2:** Hydrothermal cycle schedule used in own tests.

Cycle Stage	Activity	Duration (s)	Remarks
1	Pumping cooled liquid into the sample vessel	35	Transporting fluid to the sample
2	Holding cooled liquid—*t*_min	30	Maintaining the minimum temperature
3	Pumping out cooled liquid	35	Removing cold liquid
4	Pause	0.5	Short transition time
5	Pumping heated liquid into the sample vessel	35	Transporting fluid to the sample
6	Holding heated liquid—t_max	30	Maintaining maximum temperature
7	Pumping out heated liquid	35	Removing warm liquid
8	Pause	0.5	Short transition time

**Table 3:** Test results (WOF) for composite ageing (2 weeks in 37 °C artificial saliva), TC (10^4^ thermocycles +10/+65 °C in water).

Material	Conditioning	N	Mean	Std. Dev.	m	Scale Parameter (W_0_, W_F_)	dL (Mean)
			J 10^−3^				%
**WOF (TFS test)**							
**Z550**	Ageing	15	7.6	2.25	2.55	5.36	1.58
	ageing + TC	15	2.37	1.52	1.79	2.67	0.70
**Ex-mhyb(P)**	Ageing	15	2.70	1.07	2.79	3.04	0.73
	ageing + TC	15	0.99	0.33	3.60	1.10	0.42
**F_Flow_**	Ageing	15	13.65	4.85	3.14	15.26	2.3
	ageing + TC	15	1.90	0.98	1.97	2.17	0.72
**Ex-flow(P)**	Ageing	15	3.54	1.82	2.45	3.99	1.09
	ageing + TC	15	1.47	0.78	2.55	1.65	0.71
**WOF (BFS test)**							
**Z550**	Ageing	20	5.99	1.79	3.47	6.69	8.8
	ageing + TC	20	3.03	0.86	3.99	3.34	6.1
**Ex-mhyb(P)**	Ageing	20	8.28	4.67	2.24	9.35	12.3
	ageing + TC	20	5.93	1.37	4.86	6.47	8.5
**F_Flow_**	Ageing	20	12.46	2.46	5.60	13.47	17.7
	ageing + TC	20	7.68	2.85	2.84	8.65	14.2
**Ex-flow(P)**	Ageing	20	10.53	2.50	3.96	11.67	15.4
	ageing + TC	20	3.21	1.08	3.37	3.59	7.8
**WOF (CS test)**							
**Z550**	Ageing	20	2894.32	409.66	7.73	3073	21.3
	ageing + TC	20	3002.74	457.82	6.82	3212	21.3
**Ex-mhyb(P)**	Ageing	20	3250.36	483.55	7.27	3461.2	20.9
	ageing + TC	20	3342.23	348.78	10.714	3497.6	21.3
**F_Flow_**	Ageing	20	3427.08	536.13	6.5	3675.3	23.7
	ageing + TC	20	3064.86	240.74	14.045	3176.6	23.3
**Ex-flow(P)**	Ageing	20	3458.10	404.65	9.64	3633	25.2
	ageing + TC	20	4041.08	1109.72	4.34	4437.3	30.0

## Data Availability

The original contributions presented in this study are included in the article. Further inquiries can be directed to the corresponding author.

## Abbreviations

The following abbreviations are used in this manuscript:

RBCs	Resin-Based Composites
K_IC_	Plane Strain Fracture Toughness
WOF	Work of Fracture
TFS	Three-Point Flexure Strength
BFS	Biaxial Flexure Strength
CS	Compression Strength
SEM	Scanning Electron Microscopy
SENB	Single-Edge Notch Beam
ISO	International Organization for Standardization
ASTM	American Society for Testing and Materials

## References

[1] Yu H., Yao J., Du Z., Guo J., Lei W. (2024). Comparative evaluation of mechanical properties and color stability of dental resin composites for chairside provisional restorations. Polymers.

[2] Temizci T., Bozoğulları H.N. (2024). Effect of thermocycling on the mechanical properties of permanent composite-based CAD-CAM restorative materials produced by additive and subtractive manufacturing techniques. BMC Oral Health.

[3] Walczak A., Niewczas A.M., Pieniak D., Rogula-Kozłowska W., Kordos P., Przystupa K., Łukaszewicz A., Popovych V. (2023). Effect of the structure and hydrothermal conditions on the strength of polymer-ceramic composites. J. Achiev. Mater. Manuf. Eng..

[4] Pieniak D., Niewczas A. (2012). Phenomenological evaluation of fatigue cracking of dental restorations under conditions of cyclic mechanical loads. Acta Bioeng. Biomech..

[5] Zhou Z.R., Zheng J. (2008). Tribology of dental materials: A review. J. Phys. D Appl. Phys..

[6] He L.H., Swain M.V. (2008). Understanding the mechanical behaviour of human enamel from its structural and compositional characteristics. J. Mech. Behav. Biomed. Mater..

[7] Stewardson D.A., Shortall A.C., Marquis P.M. (2010). The effect of clinically relevant thermocycling on the flexural properties of endodontic post materials. J. Dent..

[8] Atay A., Palazli Z., Gürdal I., Üşümez A. (2019). Color change of different dual-cure resin cements after thermocycling. Odovtos Int. J. Dent. Sci..

[9] Khan A., Qureshi B., Qureshi A., Imtiaz Y., Qadeer S. (2018). Correlation of salivary characteristics with high risk of dental caries: A clinical investigation. Future Dent. J..

[10] Ayatollahi M.R., Yahya M.Y., Karimzadeh A., Nikkhooyifar M., Ayob A. (2015). Effects of temperature change and beverage on mechanical and tribological properties of dental restorative composites. Mater. Sci. Eng. C.

[11] Musanje L., Darvell B.W. (2004). Effects of strain rate and temperature on the mechanical properties of resin composites. Dent. Mater..

[12] Ferracane J.L., Palin W.M., Vallittu P. (2013). Effects of particulate filler systems on the properties and performance of dental polymer composites. Non-Metallic Biomaterials for Tooth Repair and Replacement.

[13] Krzyzak A., Racinowski D., Szczepaniak R., Kosicka E. (2023). An assessment of the reliability of CFRP composites used in nodes of friction after impact of UV-A impacts and thermal shocks. Eksploat. Niezawodn..

[14] Thadathil Varghese J., Babaei B., Farrar P., Prentice L., Prusty B.G. (2022). Influence of thermal and thermomechanical stimuli on a molar tooth treated with resin-based restorative dental composites. Dent. Mater..

[15] Hickel R., Peschke A., Tyas M., Mjör I., Bayne S., Peters M., Hiller K.A., Randall R., Vanherle G., Heintze S.D. (2010). FDI World Dental Federation—Clinical criteria for the evaluation of direct and indirect restorations: Update and clinical examples. J. Adhes. Dent..

[16] Demarco F.F., Cenci M.S., Montagner A.F., de Lima V.P., Correa M.B., Moraes R.R., Opdam N.J.M. (2023). Longevity of composite restorations is definitely not only about materials. Dent. Mater..

[17] Al-Ibrahim I., Shono N., Al-Saud L., Al-Nahedh H. (2025). Five years of restorative resin-based composite advancements: A narrative review. BMC Oral Health.

[18] Da Rosa Rodolpho P.A., Rodolfo B., Collares K., Correa M.B., Demarco F.F., Opdam N.J., Cenci M.S., Moraes R.R. (2022). Clinical performance of posterior resin composite restorations after up to 33 years. Dent. Mater..

[19] Demarco F.F., Collares K., Correa M.B., Cenci M.S., Moraes R.R., Opdam N.J.M. (2017). Should my composite restorations last forever? Why are they failing?. Braz. Oral Res..

[20] Holban A.-M., Farcasiu C., Andrei O.-C., Grumezescu A.M., Farcasiu A.-T. (2021). Surface modification to modulate microbial biofilms—Applications in dental medicine. Materials.

[21] Timothy C.N., Antony S.D.P. (2021). Prevalence of secondary caries among different restorations. J. Res. Med. Dent. Sci..

[22] Freeman R., Varanasi S., Meyers I.A., Symons A.L. (2012). Effect of air abrasion and thermocycling on resin adaptation and shear bond strength to dentin for an etch-and-rinse and self-etch resin adhesive. Dent. Mater. J..

[23] Kim K.-L., Namgung C., Cho B.-H. (2013). The effect of clinical performance on the survival estimates of direct restorations. Restor. Dent. Endod..

[24] Fischer J., Zbären C., Stawarczyk B., Hämmerle C.H.F. (2009). The effect of thermal cycling on metal-ceramic bond strength. J. Dent..

[25] Łagocka R., Granat M., Lewusz-Butkiewcz K., Tomasik M., Lipski M. (2023). The effect of thermal cycling on the surface roughness of nanohybrid and high-viscosity bulk-fill resin-based composites. Pomeranian J. Life Sci..

[26] Sun J., Jiang J., Huang Z., Ma X., Shen T., Pan J., Bi Z. (2025). Smart biomaterials in restorative dentistry: Recent advances and future perspectives. Mater. Today Bio.

[27] Li Q., Zhan N., Ng T., Swain M.V., Wan B., Jian Y., Wang X., Zhao K. (2024). The influence of hygroscopic expansion of resin supporting dies on the fracture resistance of ceramic restorations during thermal cycling. Dent. Mater..

[28] Filemban H., Bhadila G., Wang X., Melo M.A.S., Oates T.W., Hack G.D., Lynch C.D., Weir M.D., Sun J., Xu H.H.K. (2022). Effects of thermal cycling on mechanical and antibacterial durability of bioactive low-shrinkage-stress nanocomposite. J. Dent..

[29] Lohbauer U., Belli R., Ferracane J.L. (2013). Factors involved in mechanical fatigue degradation of dental resin composites. J. Dent. Res..

[30] Kruzic J.J., Arsecularatne J.A., Tanaka C.B., Hoffman M.J., Cesar P.F. (2018). Recent advances in understanding the fatigue and wear behavior of dental composites and ceramics. J. Mech. Behav. Biomed. Mater..

[31] Pieniak D., Niewczas A.M., Niewczas A., Bieniaś J. (2011). Analysis of survival probability and reliability of tooth-composite filling system. Eksploat. I Niezawodn.–Maint. Reliab..

[32] Pieniak D., Niewczas A.M., Kordos P. (2012). Influence of thermal fatigue and ageing on microhardness of polymer-ceramic composites for biomedical applications. Eksploat. Niezawodn..

[33] Szczesio-Wlodarczyk A., Barszczewska-Rybarek I.M., Chrószcz-Porębska M.W., Kopacz K., Sokolowski J., Bociong K. (2023). Can modification with urethane derivatives or the addition of an anti-hydrolysis agent influence the hydrolytic stability of resin dental composite?. Int. J. Mol. Sci..

[34] Versluis A., Tantbirojn D., Pintado M.R., DeLong R., Douglas W.H. (2004). Residual shrinkage stress distributions in molars after composite restoration. Dent. Mater..

[35] Ilie N., Hilton T.J., Heintze S.D., Hickel R., Watts D.C., Silikas N., Stansbury J.W., Cadenaro M., Ferracane J.L. (2017). Academy of Dental Materials guidance—Resin composites: Part I—Mechanical properties. Dent. Mater..

[36] Sideridou I., Achilias D.S., Kyrikou E. (2004). Thermal expansion characteristics of light-cured dental resins and resin composites. Biomaterials.

[37] Nowacki W. (1986). Thermoelasticity.

[38] Marandu S.I., Gu G., Bicker R. (2015). Experimental and analytical study of surface fatigue life in dental composites. J. Compos. Mater..

[39] Niewczas A.M. Application of censored survival data for evaluation of the dental restorations replacement criteria. Proceedings of the International Conference on Risk Analysis (ICRA 4).

[40] Leibrock H., Degenhart M., Behr M., Rosentritt M., Handel G. (1999). In vitro study on the effect of thermo- and load-cycling on the bond strength of porcelain repair systems. J. Oral Rehabil..

[41] Xu H., Eichmiller F., Smith D., Schumacher G., Giuseppetti A., Antonucci J. (2002). Effect of thermal cycling of whiskers-reinforced dental resin composites. J. Mater. Sci. Mater. Med..

[42] Reis A., Loguercio A.D., Kraul A., Matson E. (2004). Reattachment of fractured teeth: A review of literature regarding techniques and materials. Oper. Dent..

[43] Baudin C., Osorio R., Toledano M., de Aza S. (2009). Work of fracture of a composite resin: Fracture-toughening mechanisms. J. Biomed. Mater. Res. A.

[44] Bruschi-Alonso R.C., Alonso R.C., Correr G.M., Alves M.C., Lewgoy H.R., Sinhoreti M.A., Puppin-Rontani R.M., Correr-Sobrinho L. (2010). Reattachment of anterior fractured teeth: Effect of materials and techniques on impact strength. Dent. Traumatol..

[45] Wu Y.-R., Chang C.-W., Chang K.-C., Lin D.-J., Ko C.-L., Wu H.-Y., Chen W.-C. (2019). Effect of micro-/nano-hybrid hydroxyapatite rod reinforcement in composite resins on strength through thermal cycling. Polym. Compos..

[46] Cho K., Rajan G., Farrar P., Prentice L., Prusty B.G. (2022). Dental resin composites: A review on materials to product realizations. Compos. Part B Eng..

[47] Zubrzycki J., Klepka T., Marchewka M., Zubrzycki R. (2022). Tests of dental properties of composite materials containing nanohybrid filler. Materials.

[48] Katta P.K. (2025). Strength of bond between adhesives and low-viscosity bulk-fill composites utilizing 10-methacryloyloxydecyl dihydrogen phosphate (10-MDP). Biomed. Pharmacol. J..

[49] Dukić W., Majić M., Prica N., Oreški I. (2021). Clinical evaluation of flowable composite materials in permanent molars small class I restorations: 3-year double blind clinical study. Materials.

[50] 3M™ Filtek™ Z550—A Universal Nanohybrid Composite Material for Fillings. It Offers High Strength, Aesthetic Appearance and Is Easy to Polish. 3M Poland. https://www.solventum.com/pl-pl/home/f/b00007982/#product-specifications.

[51] 3M™ Filtek™ Ultimate Flow—A Universal, Low-Viscosity, Light-Cured Composite Material for a Variety of Applications in Dentistry. 3M Poland. https://www.solventum.com/pl-pl/home/f/b00007942/#product-specifications.

[52] (2019). Dentistry—Polymer-Based Restorative Materials.

[53] (2024). Dentistry—Ceramic Materials.

[54] (2020). Standard Test Method for Linear-Elastic Plane-Strain Fracture Toughness of Metallic Materials.

[55] (2018). Testing of Plastics—Bending Test and Impact Test on Dynstat Test Specimens.

[56] (2020). Dentistry—Corrosion Test Methods for Metallic Materials.

[57] Szczesio-Wlodarczyk A., Sokolowski J., Kleczewska J., Bociong K. (2020). Ageing of dental composites based on methacrylate resins—A critical review of the causes and method of assessment. Polymers.

[58] Çakmak G., Subaşı M.G., Yilmaz B. (2021). Effect of thermocycling on the surface properties of resin-matrix CAD-CAM ceramics after different surface treatments. J. Mech. Behav. Biomed. Mater..

[59] Niewczas J., Zamościńska J., Krzyżak A., Pieniak D., Walczak A., Bartnik G. (2017). Influence of fibre reinforcement on selected mechanical properties of dental composites. Acta Bioeng. Biomech..

[60] Pieniak D. (2018). Initiation and tolerance of macro-damage of first ply (FBF) in a process of damaging of hybrid multi-ply structures due to reinforcement architecture. Adv. Mater. Sci..

[61] Thomaidis S., Pappa E., Antoniadou M. (2025). Fracture toughness of CAD/CAM resin-based materials vs. direct composite resins: A Scoping review. Appl. Sci..

[62] Perez N. (2017). Fracture Mechanics.

[63] Jargalsaikhan U., Leung N., Wan H., Su B., Sui T. (2025). In situ investigation of the fracture toughening mechanisms of bioinspired dental ceramic composites with different compliant polymer phases. Dent. Mater..

[64] Vignesh K., Kandaswamy E., Muthu M. (2020). A comparative evaluation of fracture toughness of composite resin vs Protemp 4 for use in strip crowns: An in vitro study. Int. J. Clin. Pediatr. Dent..

[65] Thomaidis S., Kakaboura A., Mueller W.D., Zinelis S. (2013). Mechanical properties of contemporary composite resins and their interrelations. Dent. Mater..

[66] Hernández Ramos M.M., Piña Monarrez M.R., Barraza Contreras J.M., Monclova Quintanaz O. (2025). Weibull reliability methodology based on cumulated vibration damage. Eksploat. Niezawodn..

[67] Latoui R., Bouzid D., Boyron O. (2025). Dental composites: A comprehensive review on formulation, properties and recent developments. Polym. Int..

[68] Shangguan A., Feng N., Fei R., Hei X., Jin Y., Mu L. (2024). Reliability assessment of competitive failure systems based on three parameter Weibull distribution and Wiener process. Eksploat. Niezawodn..

[69] Hamouda I.M., Elkader H.A. (2012). Evaluation the mechanical properties of nanofilled composite resin restorative material. J. Biomater. Nanobiotechnol..

[70] De Souza J.A., Goutianos S., Skovgaard M., Sørensen B.F. (2001). Fracture resistance curves and toughening mechanisms in polymer based dental composites. J. Mech. Behav. Biomed. Mater..

[71] Althaqafi K.A. (2025). Performance of direct and indirect onlay restorations for structurally compromised teeth. J. Prosthet. Dent..

[72] Siddiqui M.A.S., Hossain M.A.M., Ferdous R., Rabbi M.S., Yeasar Abid S.M.S. (2025). An extensive review on bibliometric analysis of carbon nanostructure reinforced composites. Results Mater..

[73] Walczak M., Szala M., Pieniak D. (2022). Effect of water absorption on tribological properties of thermoplastics matrix composites reinforced with glass fibres. Adv. Sci. Technol. Res. J..

[74] Ferracane J.L. (2006). Hygroscopic and hydrolytic effects in dental polymer networks. Dent. Mater..

[75] Kang T.W., Park K., Kim M.S. (2026). Advances in stimuli-responsive polymers for biomedical and environmental applications. Mater. Sci. Eng. R Rep..

[76] Afzal A., Shaker K. (2026). Hydrothermal degradation of polymer nanocomposites. Aging and Degradation of Polymer Nanocomposites.

[77] Troha L., Šraj B., Par M., Simeon P., Haugen H.J., Tarle Z., Marciuš M., Marovic D. (2026). Filler amount influences long-term mechanical stability of experimental dental composites. Dent. Mater..

[78] Huang X., Zhang D., He X., Yang H., Yu Y., Yang X., Cai Q. (2026). Piezoelectric-pyroelectric inorganic filler synergistically endows dental resin composite with long-term self-bactericidal potential. Chem. Eng. J..

[79] Flottes Y., Smail Y., Palomino-Durand C., Attal J.-P., Ceinos R., François P., Dursun E. (2026). Properties of 3D printed resins for definitive dental restorations: A systematic review. J. Prosthet. Dent..

[80] Vennapusa C.S.R., Eluru A.K. (2026). Experimental studies on environmental constraints and remedial measures for environmental sustainability: A review. Sustain. Cities Soc. Adv..

[81] Vignesh J., Ramesh B., Xavier J.R. (2025). A comprehensive review of materials, processing, and performance of nano-doped engineered geopolymer composites for construction applications. Case Stud. Constr. Mater..

[82] Selvaraj V., Saravanan R., Vikram N.R., Gopalakrishnan U.R., M R. (2025). Exploring the sources and routes of micro- and nanoplastics from dental products and materials: Their impact on human health—A systematic review. Next Res..

[83] Ahmed I.U. (2026). Role of nanotechnology in remediation of microplastics from aquatic environments. Remediation Technologies for Microplastics in Aquatic Environments.

[84] Mann R.S., Ruse N.D. (2022). Fracture toughness of conventional, milled and 3D printed denture bases. Dent. Mater..

[85] Johansson L., Raymond Y., Labay C., Mateu-Sanz M., Ginebra M.-P. (2024). Enhancing the mechanical performance of 3D-printed self-hardening calcium phosphate bone scaffolds: PLGA-based strategies. Ceram. Int..

[86] Henry Dusim G.A., Muhamad F., Lai K.W. (2025). Enhancing calcium phosphate cements: A review of bacterial cellulose (BC) and other biopolymer reinforcements for biomedical applications. Biomater. Adv..

[87] Tonin B.S.H., Kava L.E., Vilela H.S., Palma-Dibb R.G., Braga R.R. (2026). Predictive modeling of composite fracture toughness using machine learning. Dent. Mater..

[88] Thadathil Varghese J., Cho K., Raju, Farrar P., Prentice L., Prusty B.G. (2023). Effect of silane coupling agent and concentration on fracture toughness and water sorption behaviour of fibre-reinforced dental composites. Dent. Mater..

[89] Aliha M.R.M., Pietras D., Rajabi-Kafshgar A., Sadowski T. (2026). Fracture toughness and fracture energy of polymeric concrete with variable mixtures designed by the L32 Taguchi method and statistical analysis of pore sizes in the fracture surface. Constr. Build. Mater..

[90] Wu Y.-R., Chang C.-W., Ko C.-L., Wu H.-Y., Chen W.-C. (2017). The morphological effect of calcium phosphates as reinforcement in methacrylate-based dental composite resins on mechanical strength through thermal cycling. Ceram. Int..

[91] Islam M.A., Hossain N., Hossain S., Khan F., Hossain S., Arup M.M.R., Chowdhury M.A., Rahman M.M. (2025). Advances of hydroxyapatite nanoparticles in dental implant applications. Int. Dent. J..

[92] Sahoo N., Ghosh A., Khan M.D.A., Ray B.C., Patel P., Das B., Sahoo S.P., Ranjan P., Shrivastava P., Msomi V. (2026). Functionally graded materials: Development, processing techniques, and emerging applications—A comprehensive review. Mater. Today Commun..

[93] Heintze S.D., Zellweger G., Zappini G. (2007). The relationship between physical parameters and wear of dental composites. Wear.

[94] Saini S., Meena A., Yadav R., Patnaik A. (2023). Investigation of physical, mechanical, thermal, and tribological characterization of tricalcium phosphate and zirconia particulate reinforced dental resin composite materials. Tribol. Int..

[95] Casucci A., Maniewicz S., Spyraki F., Müller F., Chebib N. (2025). Occlusal wear in dental prostheses milled from a two-colored shell-geometry disk: A prospective clinical pilot study. Digit. Dent. J..

[96] Arslan Acicbe B., Deniz S., Dönmez M.B., Diken Türksayar A.A., Demirel M. (2026). Wear, fracture strength, and reliability of three-unit definitive fixed dental prostheses fabricated with different vat polymerization methods. J. Dent..

[97] Ferracane J.L., Berge H.X., Condon J.R. (1998). In vitro aging of dental composites in water—Effect of degree of conversion, filler volume, and filler/matrix coupling. J. Biomed. Mater. Res..

[98] Delaviz Y., Finer Y., Santerre J.P. (2014). Biodegradation of resin composites and adhesives by oral bacteria and saliva: A rationale for new material designs that consider the clinical environment and treatment challenges. Dent. Mater..

[99] Yang K., Nugraha A.P., Chen J., Yang H., Wang J., Sáenz J.R.V., Hong G. (2025). Utilization of cellulose nanofiber in dental applications: A systematic review of in vitro evidence. Jpn. Dent. Sci. Rev..

[100] Peled Y., Ragheai A., Gouveia Z., Stewart C.A., Zargaran S., Elebyary O., Sun C., Glogauer M., Finer Y. (2025). Pathways of neutrophil enzymatic degradation of resin-based composites and adhesives. Acta Biomater..

[101] Lagowski M., Gouveia Z., Yang M., Finer Y., Santerre J.P. (2024). Synthesis and challenges of fluorinated divinyl urethane monomers as a strategy for masking hydrolytic sensitive methacrylate groups in resin composites. Dent. Mater..

[102] Gouveia Z., Peled Y., Rahiminejad R., Finer Y., Santerre J.P. (2026). Immune and microbial cellular interactions with contemporary and alternative resin-based dental restorative materials. J. Dent..

